# Effects of earplugs and eye masks on nocturnal sleep, melatonin and cortisol in a simulated intensive care unit environment

**DOI:** 10.1186/cc8965

**Published:** 2010-04-18

**Authors:** Rong-fang Hu, Xiao-ying Jiang, Yi-ming Zeng, Xiao-yang Chen, You-hua Zhang

**Affiliations:** 1 School of Nursing, Fujian Medical University, Jiaotong Road 88, Fuzhou, 350004, PR China; 2 Sleep-Breath Disorders Center, Second Affiliated Hospital, Zhongshan Road 34, Quanzhou, 362000, PR China; 3 Department of Nuclear Medicine, Fujian Province Hospital, East Street 134, Fuzhou, 350001, PR China

## Abstract

**Introduction:**

Environmental stimulus, especially noise and light, is thought to disrupt sleep in patients in the intensive care unit (ICU). This study aimed to determine the physiological and psychological effects of ICU noise and light, and of earplugs and eye masks, used in these conditions in healthy subjects.

**Methods:**

Fourteen subjects underwent polysomnography under four conditions: adaptation, baseline, exposure to recorded ICU noise and light (NL), and NL plus use of earplugs and eye masks (NLEE). Urine was analyzed for melatonin and cortisol levels. Subjects rated their perceived sleep quality, anxiety levels and perception of environmental stimuli.

**Results:**

Subjects had poorer perceived sleep quality, more light sleep, longer rapid eye movement (REM) latency, less REM sleep when exposed to simulated ICU noise and light (*P *< 0.05). Nocturnal melatonin (*P *= 0.007) and cortisol secretion levels (*P *= 0.004) differed significantly by condition but anxiety levels did not (*P *= 0.06). Use of earplugs and eye masks resulted in more REM time, shorter REM latency, less arousal (*P *< 0.05) and elevated melatonin levels (*P *= 0.002).

**Conclusions:**

Earplugs and eye masks promote sleep and hormone balance in healthy subjects exposed to simulated ICU noise and light, making their promotion in ICU patients reasonable.

## Introduction

Sleep disruption is common in ICU patients and has been characterized by several studies using polysomnography (PSG) [1-3]. Adverse consequences of sleep disruption include impaired immune function, decreased inspiratory muscle endurance, negatively affected weaning from mechanical ventilation, and a possible association with delirium and severe morbidity [4,5]. The causes of sleep disruption in the ICU are multifactorial. The ICU environment is thought to be an important factor in sleep disruption [6].

Numerous studies have found excessive noise levels in the ICU, often with nighttime peaks of more than 80 dB(A) [5,7]. In addition, subjective and objective studies both demonstrate that patients have been disturbed by ICU noise [1,8-10]. Light exposure is another important sleep disruptor in ICU settings. Reported nocturnal illumination in ICUs varies widely, with mean maximum levels of 5 to 1400 lux [5,11]. Light exposure is the primary external cue for circadian rhythm. In addition, nocturnal melatonin secretion can be acutely suppressed by light, and 100 lux is sufficient to impact nocturnal melatonin secretion [12]. Throughout the past decade, evidence has been accumulating for the altered secretion of melatonin in ICU patients. ICU patients suffer from a severe lack of sleep associated with loss of the nocturnal melatonin secretion pattern [13,14]. Therefore, effective interventions to promote sleep in ICU patients are urgently needed.

Despite many claims that the use of noise reduction and lighting practice in an intensive care environment may improve the patient's sleep quality, there have been few objective studies to evaluate the effects of these interventions [15-17]. Most research in this area has focused purely on noise reduction and not explored the combined effects of ICU noise and light factors on physiological and psychological outcomes, including sleep architecture, perceived sleep quality and hormone secretion (melatonin and cortisol). No studies have yet evaluated the effects of earplugs and eye masks on the sleep of ICU patients as measured by PSG and hormone secretion.

We hypothesized that patients' sleep is disrupted by the noise and light in the ICU, accompanied by impaired nocturnal melatonin secretion and elevated cortisol secretion. Earplugs and eye masks worn during exposure to a simulated ICU environment may improve sleep and protect nocturnal melatonin and cortisol secretion. To test this hypothesis, an experimental study was conducted in a sleep laboratory.

## Materials and methods

### Research design

This study used a repeated measures design. Four nocturnal nine-hour (10:00 p.m. to 7:00 a.m.) periods of sleep were measured, including adaptation, baseline, exposure to recorded ICU noise and light (NL), and NL plus use of earplugs and eye masks (NLEE). All subjects (n = 14) underwent a total of four overnight PSG.

**Figure 1:**
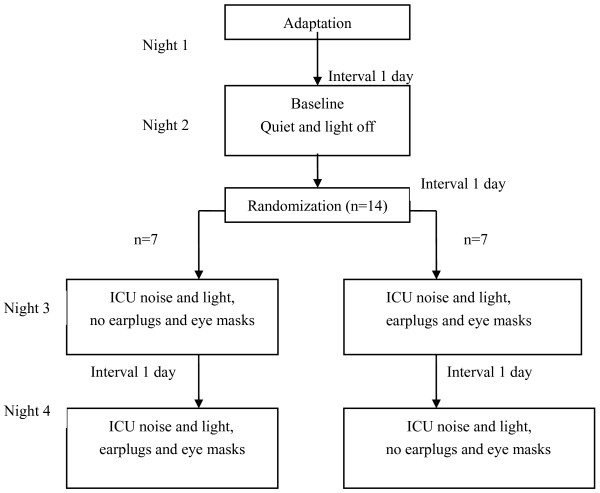
**Study design**.

To minimize order effects, earplugs and eye masks were randomly worn on either the third (n = 7) or fourth (n = 7) night (Figure 1). For each subject, study nights were spaced one day apart to avoid delay effects. Subjects were asked to keep a sleep diary to record their rest, activity and diet during the study period.

Subjects were asked to provide two nocturnal (10:00 p.m. to 7:00 a.m.) urine samples, during base, NL and NLEE nights. Urine levels of melatonin sulfate and cortisol were determined by ELISA and radioimmunoassay (RIA), respectively.

All subjects completed a sleep scale and the Chinese version of the Spielberger State Anxiety Inventory (SAI) [18] at 7:30 a.m. after every experimental night to record perceived sleep quality and anxiety levels, respectively.

The study was performed at the Sleep-breath Disorders Center at the Second Affiliated Hospital, Fujian, China. The study design was approved by the research ethics boards of the hospital and the Fujian Medical University.

### Subjects

Subjects were included in the study if they were nonsmokers, older than 18 years of age, had no hearing problem as determined by a hearing screening test, had no sleep disorders, scores of 7 or less on the Pittsburgh Sleep Quality Index (PSQI), had no history of night-shift work in the past three years and agreed to abstain from caffeine and alcohol for 12 hours prior to each study night. All provided written informed consent before being enrolled in the study.

Subjects were excluded if PSG on the adaptation night found any sleep disorder including sleep apnea, narcolepsy, chronic insomnia or restless leg syndrome. Female subjects were excluded while they were menstruating.

Subjects were recruited by advertisements posted at the Second Affiliated Hospital. Fourteen subjects were enrolled in the study. Each was paid 200 Yuan Renminbi at the end of the study.

### Instruments

#### Recorded ICU noise

ICU noise was continuously monitored for 24 hours using a sound meter, model AWA5610D (AWAI, Hangzhou, China) in five ICU environments: a surgical ICU (SICU), a coronary care unit (CCU), a cardiac surgical ICU (CSICU) and two medical ICUs (MICU). All had noise levels far exceeding the 20 dB(A) at nighttime recommended by the Guidelines of the Chinese Association of Critical Care Medicine (2006)[19]. The SICU was the loudest. The mean (standard deviation) noise value in the SICU was 70.1 ± 11.9 dB(A), the peak noise level recorded was 95.3 dB(A), and the minimal noise value was 51.4 dB(A). Thereafter, ambient noise of the SICU and CSICU were recorded digitally during a typical weeknight shift and stored on computer for playback in the sleep laboratory. The sound recording equipment, model ICD-P320 (Sony Inc., Tokyo, Japan) was positioned at the bed of patients receiving mechanical ventilation. Simultaneous sound meter readings were taken to ensure similar noise levels during playback in the sleep laboratory.

#### Lighting conditions

Nighttime illumination in five ICU settings and the sleep laboratory were monitored by a light detector, model TES1332 (Taiwantes, Shenzen, China). In all settings, light was provided by ceiling fluorescent lights. The light detector was placed as close as possible to the head of the bed of a patient receiving mechanical ventilation, but not so as to interfere with patient care. Light measurements were taken every hour for 24 hours. Five ICUs maintained high mean night light levels ranging between 50 and 238.6 lux, with highest mean nighttime light levels in the SICU and CSICU. Mean nighttime light levels in the sleep laboratory measured 100 lux with the light on, and 5 lux with it off and the door to the hallway shut. Therefore, the study used 100 lux to simulate the ICU lighting condition.

#### Earplugs and eye masks

Subjects were instructed to wear earplugs with 29 dB noise reduction rating (3 M Corporation, Beijing, China) and eye masks during experimental night NLEE. Subjects chose from three types of eye masks provided.

#### Polysomnography

Sleep was assessed by PSG using the Polysmith 2003 sleep acquisition and analysis system (Neurotronics, Gainesville, FL, USA). The standard procedure for sleep measurement described by Rechtschaffen and Kales [20] was followed. Subjects were hooked up to record electroencephalogram (EEG), eye movement and sub-mental electromyogram (CHin EMG) in the sleep laboratory. During NL and NLEE nights, recorded ICU noise was played and fluorescent lights turned on. A sound meter was placed at the head of the subject's bed and the recording time synchronized with the sound meter to ensure playback in a similar range of decibels to that recorded. Electrode impedances were within acceptable limits (<10kQΩ). PSG equipment was located outside the subject's room. Sleep variables (sleep period time, sleep efficiency index, sleep onset latency, rapid eye movement (REM) latency, arousal index and percentage of sleep in REM, stage one, two and three) were scored manually by two scorers independently who were unaware of the experimental conditions, according to standardized criteria [20,21].

### Melatonin and cortisol

Nocturnal urine was collected between 10 p.m. and 7 a.m. on baseline, NL and NLEE nights. The containers were wrapped with black plastic to protect the urine from light. The amount was recorded and two samples of each 2 ml were frozen to -20°C for later analysis. Concentrations of 6-sulphatoxymelatonin (6-SMT), the stable metabolite of melatonin, were measured by enzyme-linked immunometric assay (IBL, Hamburg, Germany) in duplicate. Concentration of cortisol, a stress-related hormone, was measured in another urine sample by RIA (Beijing North Institute of Biological Technology, Beijing, China).

### Subjective measurements

Subjective sleep quality was assessed by a visual analog scale developed by the researchers based on previous scales [22]. Subjects evaluated their sleep quality on a scale of 0 to 10 (0 = excellent, 10 = poor) at 7:30 a.m. on the morning after every experimental night, with a higher score indicating poorer habitual sleep quality.

State anxiety level was assessed at 7:30 a.m. on the morning after every experimental nights. In our study, Spielberger State Anxiety Inventory (SAI) was chosen because it provides evaluation of state anxiety levels, namely a temporary unpleasant emotional arousal in the face of threatening demands or dangers [23]. Subjects rated their feelings of anxiety on a four-point scale ranging from one (almost never anxious) to four (almost always anxious), a higher score indicating a higher anxiety level.

On the morning after the NL night, subjects were asked to assess the effects of simulated ICU noise and light on sleep disruption, using a five-point scale ranging from one (no disruption) to five (significant disruption).

Subjects were asked to evaluate the comfort, effectiveness and ease of use of earplugs and eye masks on the morning after the NLEE night, using a five-point scale ranging from one (very uncomfortable, very unhelpful, very awkward) to five (very comfortable, very helpful, very easy to use) with low scores indicating a less pleasant experience.

### Statistical analysis

Data were analyzed using SPSS version 16.0 (SPSS Inc., Chicago, IL, USA). Data for the adaptation night were excluded from analysis because the first night of sleep in a sleep laboratory room with unfamiliar surroundings differs from sleep on subsequent nights [24]. All data were expressed as mean ± standard deviation. One-way repeated measures analysis of variance (ANOVA) were used to determine differences in sleep variables, 6-SMT and cortisol concentrations, perceived sleep quality and anxiety levels during the three nights of the experiment. Paired student's *t*-test or non-parametric Wilcoxon's rank sum test were performed to evaluate the effect of earplugs and eye masks on sleep variables and hormones secretion during exposure to simulated ICU sound and light where appropriate. Paired sample test was also used to analyze differences in sound levels between NL and NLEE nights. An alpha of 0.05 was considered significant.

## Results

Fifteen healthy volunteers were recruited. One was excluded due to evidence of significant insomnia. A total of 14 subjects (8 females and 6 males, aged 21 to 70 years, mean 31.07 ± 15.64 years) completed the study. The earplugs and eye masks were applied easily and remained intact during NLEE nights.

### Sleep architecture

**Figure 2:**
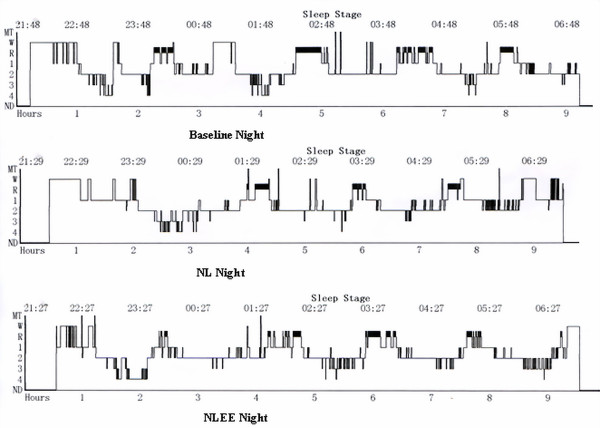
**Example of sleep histograms from the same subject**. Less rapid eye movement (REM) time, longer REM latency as exposure to recorded ICU noise and light. NL, recorded ICU noise and light exposure; NLEE, recorded ICU noise and light, subjects wore earplugs and eye masks.

Results of sleep variables during baseline, NL and NLEE nights are shown in Table 1. Repeated measures ANOVA showed that sleep architecture changed significantly in percentage of REM sleep (*P *= 0.03), REM latency (*P *= 0.02) and arousal index (*P *= 0.03) by condition. Contrast of sleep variables during exposure to simulated ICU environment indicated that use of earplugs and eye masks resulted in more REM sleep (*P *= 0.005), shorter REM latency (*P *= 0.013) and fewer arousals (*P *= 0.04; Figure 2).

**Table 1:** Sleep architecture and study condition for subjects (n = 14)

Variable	Baseline	NL	NLEE	ANOVA *P*	Contrast *P*
Time in bed (min)	539.7 ± 1.7	536.0 ± 15.9	536.0 ± 13.7	0.48	----
Total sleep time (min)	456.0 ± 39.9	454.7 ± 41.8	475.1 ± 33.4	0.20	0.06
Sleep efficiency Index	0.8 ± 0.1	0.8 ± 0.1	0.9 ± 0.0	0.12	0.09
REM%	10.9 ± 5.9	9.3 ± 4.3	12.9 ± 4.3	0.03	0.005
S1%	21.8 ± 10.4	23.4 ± 11.9	22.5 ± 9.7	0.80	0.67
S2%	43.9 ± 10.2	45.6 ± 10.3	43.5 ± 6.9	0.57	0.20
S3%	14.0 ± 6.8	11.6 ± 6.5	13.9 ± 5.6	0.30	0.11
Sleep onset latency (min)	22.3 ± 13.1	23.4 ± 16.6	15.4 ± 16.4	0.46	0.055
REM latency (min)	121.8 ± 47.0	146.9 ± 56.2	105.7 ± 47.0	0.02	0.013
Arousals index	13.0 ± 4.7	15.1 ± 6.2	12.2 ± 6.5	0.03	0.04

ANOVA, repeated measures analysis of variance; Contrast, paired student's test or wilcoxon's rank sum test of simulated ICU environment with and without earplugs and eye masks; NL, recorded ICU noise and light exposure; NLEE, recorded ICU noise and light, subjects wore earplugs and eye masks; REM, rapid eye movement.

### Urinary excretion of 6-SMT and cortisol

**Figure 3:**
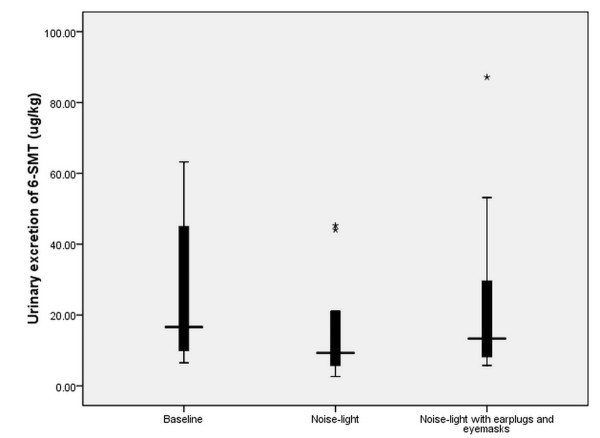
**Urinary excretion of 6-SMT for different study conditions**. Nocturnal urine 6-SMT concentration during baseline, NL and NLEE nights were 26.5 ± 20.0, 15.1 ± 13.6, and 22.3 ± 22.9 μg/kg, respectively. 6-SMT, 6-sulphatoxymelatonin; NL, recorded ICU noise and light exposure; NLEE, recorded ICU noise and light, subjects wore earplugs and eye masks.

**Figure 4:**
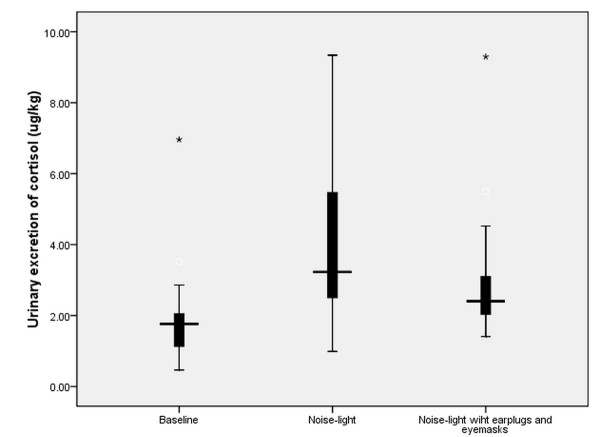
**Urinary excretion of cortisol for different study conditions**. Nocturnal urine cortisol concentration during baseline, NL and NLEE nights were 2.0 ± 1.6, 4.0 ± 2.4, and 3.2 ± 2.1 μg/kg, respectively. NL, recorded ICU noise and light exposure; NLEE, recorded ICU noise and light, subjects wore earplugs and eye masks.

Subjects' urinary excretion during baseline, NL and NLEE nights of 6-SMT and cortisol is shown in Figures 3 and 4, respectively. Differences in nocturnal urinary secretion levels of 6-SMT (*F *= 7.84, *P *= 0.007) and cortisol (*F *= 9.26, *P *= 0.004) were both significant.

Wilcoxon's rank sum test showed significant differences in urine 6-SMT levels for NL and NLEE nights (Z = -3.17, *P *= 0.002). But no difference was found in urine cortisol levels for NL and NLEE nights (Z = -1.47, *P *= 0.14).

### Subjective sleep quality and anxiety levels

The results of repeated measures ANOVA for subjective sleep quality were significant (*F *= 20.6, *P *= 0.00), but those for anxiety levels (*F *= 3.55, *P *= 0.06) were not (Table 2).

**Table 2:** Subjective assessment of sleep quality and state anxiety by condition

	Baseline night	NL night	NLEE night	ANOVA *P*	Contrast *P*
State anxiety	28.7 ± 6.3	32.5 ± 5.6	29.8 ± 6.4	0.06	0.28
Sleep quality	1.7 ± 1.3	4.1 ± 1.7	2.3 ± 1.3	0.00	0.001

ANOVA, repeated measures analysis of variance; Contrast, paired student's test or wilcoxon's rank sum test of simulated ICU environment with and without earplugs and eye masks; NL, recorded ICU noise and light exposure; NLEE, recorded ICU noise and light, subjects wore earplugs and eye masks.

Paired contrast showed use of earplugs and eye masks improved perceived sleep quality notably (*P *= 0.001).

No difference was founded in anxiety levels between the NL and NLEE nights (*P *= 0.28) by paired contrast, although SAI scores showed interesting trends that scores for NL night were highest.

### Subjective perception of ICU environment and interventions

On baseline, NL and NLEE nights, sound levels averaged 34 ± 0.6, 66.1 ± 4.2 and 66 ± 5.3 dB(A), respectively. Paired contrast revealed no significant difference in the noise level for NL and NLEE nights (*P *= 0.94). Table 3 shows the subjective perception of the effect of noise and light stimuli on sleep disruption on the NL night. Eleven subjects perceived noise factor as a little disruption but were able to fall asleep, two perceived noise as some disruption and were sometimes unable to fall sleep, and one perceived light sleep and was easily awakened by noise. There were eight, one and five subjects perceived a little disruption but were able to fall asleep, some disruption and was sometimes unable to fall asleep, and light sleep and were easily awakened by constant lighting, respectively.

**Table 3:** Subjective perception of sleep disruption (n = 14)

	No disruption	A little disruption but able to fall asleep	Sometimes unable to fall sleep	Light sleep, easy be awakened	Significant disruption, awake all night
Noise	0	11	2	1	0
Light	0	8	1	5	0

Subjects' evaluation of earplugs and eye masks is listed in Table 4. Overall, they rated the devices highly, as very comfortable, very helpful and very easy to use.

**Table 4:** Evaluation of earplugs and eye masks (n = 14)

	Helpful to sleep promotion	Comfortable	Effective for noise/light reduction	Easy to apply
Earplugs	6	6	10	11
Eye masks	8	10	13	14

## Discussion

These results support the notion that sleep and hormones are both disturbed with exposure to simulated ICU noise and light in healthy subjects. Use of earplugs and eye masks improve subjective sleep quality noticeably.

Our results confirm that subjects not only have poorer perceived sleep quality, but also suffer from sleep disruption, measured as more light sleep, longer REM latency and less REM sleep, with exposure to simulated ICU noise and light levels. The results are similar to those reported by Topf and Davis [25] and Wallace and colleagues [16]. However, our study differs in that we combined levels of noise and light in a simulated ICU environment in a sleep laboratory. 

The ICU environment is not conducive to sleep. Survey studies showed that ICU patients considered excessive noise and bright lights noxious and disruptive [26]. Freedman and colleagues reported that nurse interventions were more disruptive than noise or light [8]. Gabor and colleagues found that noise, light and patient care activities accounted for less than 30% of nocturnal arousals and awakenings [10]. Recently Cabello and colleagues reported noise accounted for less than 15% of arousals and awakenings in mechanical ventilation patients [27]. Therefore, noise has proved to be an important sleep-disruptive factor and has negative physiological and psychological effects on patients, although it may not be responsible for the majority of the sleep fragmentation. Recent emphasis has been on noise reduction and encouraging the dimming of lights overnight in ICU settings, but control of noise is not always possible and lights are always present in critical care for patient observations and patient care activities. Therefore, we hypothesized that use of earplugs and eye masks may have benefits in some ICU patients with regards to sleep disturbances.

The tolerability of these interventions is critical. Most healthy subjects rated earplugs as comfortable and easy to use [16]. In our study, six subjects rated earplugs as comfortable and 10 rated eye masks as comfortable, and all subjects used them easily and kept them intact during the study nights. However, previous studies showed that some ICU patients were unwilling to use the earplugs and/or eye masks because they found the interventions uncomfortable [28]. Some patients commented that there was a feeling of heat, tightness, sore ears, claustrophobia and still being able to hear when using earplugs [15,29]. The reasons for this may include improper insertion, individual variability in sensitivity or anatomy of the ears, unsuitable type of earplugs and eye masks and the anxious state of patients. Future studies should consider the sleep intervention according to patients' tolerability and explore other methods when patients can not tolerate the devices. Light disturbances may be 'blocked' by other means and exclusion of blue light at night by patients wearing glasses that filter out this light wavelength or nocturnal lighting sources without blue light may be alternatives with regard to minimizing adverse effects on the nocturnal melatonin surge. In addition, critical care nurses should patiently provide accurate instruction and assistance for use of earplugs and eye masks, which may help more patients to benefit from the use of the earplugs and eye masks to promote better sleep [29].

Previous studies consistently found ICU patients suffered from severe sleep disruption, with an increased percentage of wakefulness and stage 1 sleep and a decrease or absence of both slow wave sleep and REM sleep [1-3]. Our study indicates a significant difference in PSG of percentage of REM sleep, REM latency and arousal index between NL and NLEE. Although percentage of REM sleep (12.9%) was statistically significantly higher in the NLEE period, the absolute figure in NL (9.3%) period is still high compared with that frequently reported in ICU patient PSG studies in which REM percentage is often less than 5% [1,2]. Our study cannot completely simulate the ICU scenario; the healthy volunteers slept relatively well in a sleep laboratory while ICU patients are exposed to many other physical and psychological stressors during their acute illness, which may also contribute to their sleep disturbance.

Melatonin is the key circadian regulatory hormone in humans. Cortisol is an important stress hormone. Melatonin secretion normally increases at night and decreases in the early morning hours. In contrast, cortisol secretion falls. Both are biological markers of the circadian rhythm. Melatonin therapy has been shown to be effective in the resetting of sleep-wake cycles, the entrainment of circadian rhythms and the treatment of chronic insomnia [30,31]. The melatonin secretion pattern has been related to the sleep disturbances observed in the ICU [13,14]. Recently there has been some evidence that exogenous melatonin is effective in improving sleep in ICU patients [28,32]. Sleep intervention should be extended to a coordinated exogenous melatonin therapy with bright light [28]. Friese suggested that lighting could be coupled with shielding of patient's eyes to allow for a sufficiently lit environment so ICU staff could carry out necessary nighttime activities and minimize retinal stimulation for the patients [33]. Most previous studies evaluated the effects of earplugs, and recently two studies indicated that earplugs and eye masks were a relatively cheap way to improve sleep quality in critically ill patients [15,34]. In fact, our study found that subjects disliked light as much as noise and eye masks are better than earplugs in terms of the subject's tolerability.

In addition, our finding of significantly higher cortisol levels when exposure to ICU noise and light agreed with previous results that acute noise stress invokes the stress response and high levels of stress hormones [35].

### Limitations of the study and suggestions for future studies

Our study design has a number of limitations, which should be reviewed. First, the study was performed in a sleep laboratory with healthy subjects rather than in an ICU setting of critically ill patients, and therefore could not completely simulate the full auditory and visual experience of the ICU. Second, the study was only performed for a nine-hour nocturnal period rather than over 24 hours. The ICU patients experience circadian rhythm disturbances with sleep traversing the day and night. Therefore, an ideal study should measure the sleep in healthy volunteers lying recumbent over a 24-hour period to completely simulate the ICU scenario.

Our study combined noise and light as environmental stimuli and used earplugs and eye masks as intervention, so we were unable to measure the effect of each one separately on sleep and hormones secretion. Further studies are needed to elucidate the separate mechanisms of ICU noise and light on sleep disturbance and hormones secretion. In addition, our sample sizes were small, which limited the power of our statistical analyses. Future studies with larger and more diversity of the participants would likely support these recommendations.

## Conclusions

In summary, our results found that use of earplugs and eye masks in subjects not only improves subjective sleep quality, but also increases the amount of REM sleep and nocturnal melatonin levels in a simulated ICU environment. Our pilot study provides a reasonable basis for promoting the use of earplugs and eye masks for ICU patients. Sleep is a basic human need, sleep disruption may contribute to patient morbidity and degenerate quality of life [4,5]. Therefore, we recommend the routine use of earplugs and eye masks in all ICU patients even though some patients may be undergoing ongoing disease processes. Future studies should be designed to determine if the use of earplugs and eye masks will improve the sleep quality and ultimately benefit the clinical outcome of critically ill patients.

## Key messages

• Subjects had poorer perceived sleep quality, more light sleep, longer REM latency, and less REM sleep when exposed to simulated ICU noise and light.

• Nocturnal melatonin and cortisol secretion levels differed significantly by experimental condition.

• Use of earplugs and eye masks resulted in more REM time, shorter REM latency, less arousal and an elevation of nocturnal melatonin levels.

## Abbreviations

6-SMT: 6-sulphatoxymelatonin; ANOVA: analysis of variance; CCU: coronary care unit; CHin EMG: sub-mental electromyogram; CSICU: cardiac surgical ICU; EEG: electroencephalogram; ELISA: enzyme-linked immunosorbent assay; MICU: medical ICU; NL: recorded ICU noise and light exposure; NLEE: NL plus use of earplugs and eye masks; PSG: polysomnography; PSQI: Pittsburgh Sleep Quality Index; RAI: radioimmunoassay; REM: rapid eye movement; SICU: surgical ICU; STAI: Spielberger State-Trait Anxiety Inventory.

## Competing interests

The authors declare that they have no competing interests.

## Authors' contributions

JX and ZY designed the study, and participated in the coordination and writing of the manuscript. HR performed data collection, data entry, statistical analysis and wrote the manuscript. CX participated in the data collection, data entry, and statistical analysis. ZY was responsible for analysis of the levels of 6-SMT and cortisol.
